# Machine Learning and SHAP Value Interpretation for Predicting Hepatic Steatosis Using Vibration‐Controlled Transient Elastography

**DOI:** 10.1155/ije/3395722

**Published:** 2026-06-18

**Authors:** Zhenyan Lu, Chunqiao He, Mengyuan Wang, Wei He, Shuang Wang, Ying Xie, Xue Pan

**Affiliations:** ^1^ Medical Equipment Department, The Affiliated Hospital of Southwest Medical University, Luzhou, Sichuan, China, scu.edu.cn

**Keywords:** hepatic steatosis, machine learning, predictive value, SHAP analysis, vibration-controlled transient elastography

## Abstract

**Background:**

Hepatic steatosis (HS) is a common liver condition characterized by excessive fat accumulation in hepatocytes. In the present study, we developed a machine learning (ML)‐based predictive model for HS defined by vibration‐controlled transient elastography (VCTE). Key predictive factors were further interpreted using SHapley Additive exPlanations (SHAP).

**Methods:**

This study used data from two NHANES cycles, 2017–2020 and 2021–2023. The Boruta algorithm and LASSO regression were applied for feature selection. Six ML models, including logistic regression (LR), random forest (RF), multilayer perceptron (MLP), support vector machine (SVM), K‐nearest neighbor (KNN), and extreme gradient boosting (XGBoost), were used to build prediction models. SHAP was used to interpret model predictions and identify the most influential predictors of HS. Model performance was evaluated using area under the receiver operating characteristic curve (AUC), accuracy, sensitivity, specificity, positive predictive value, and negative predictive value.

**Results:**

A total of 12,177 participants were included in the current study. Boruta and LASSO feature selection identified 14 significant predictors, comprising age, BMI, gender, glycohemoglobin, HDL cholesterol, hemoglobin, hypertension, lymphocyte, manganese, monocyte, race, segmented neutrophils, selenium, and total cholesterol. XGBoost achieved the highest AUC (0.832), followed by LR (0.826), RF (0.825), SVM (0.818), MLP (0.792), and KNN (0.737). SHAP analysis of the XGBoost model highlighted BMI, glycohemoglobin, age, and HDL cholesterol as key contributors to HS prediction.

**Conclusion:**

The XGBoost model showed the highest discriminative ability for predicting HS, with BMI, glycohemoglobin, age, and HDL cholesterol identified as the most important predictors. This interpretable model may support early identification and risk stratification of HS.

## 1. Introduction

Hepatic steatosis (HS) refers to excess lipid accumulation within hepatocytes and has become an increasing public health concern, as it increases the risk of liver fibrosis, cirrhosis, and other adverse hepatic outcomes [1]. HS is a key phenotypic feature of nonalcoholic fatty liver disease (NAFLD), whereas nonalcoholic steatohepatitis (NASH) represents its progressive inflammatory subtype. Recently, NAFLD and NASH have been renamed metabolic dysfunction‐associated steatotic liver disease (MASLD) and metabolic dysfunction‐associated steatohepatitis (MASH), respectively [2]. It is estimated that approximately 25% of the global population has NAFLD, with the prevalence in some regions, including the United States, reaching 30%–40%, posing a substantial public health burden [3, 4]. Because early‐stage disease often lacks clear symptoms, early detection and risk assessment of HS are essential to prevent progression to severe liver disease.

Liver biopsy remains the gold standard for diagnosing HS, but its invasive nature and potential complications, such as hemorrhage, pain, and sampling variability, make it unsuitable for large‐scale population studies [5]. Various noninvasive methods have been proposed for diagnosing HS, among which vibration‐controlled transient elastography (VCTE) is widely used. VCTE assesses liver stiffness and controlled attenuation parameter (CAP) to assess hepatic fat content, providing a reliable tool for HS detection [6, 7]. VCTE is useful for detecting hepatic fat deposition. However, identifying risk factors such as diabetes, metabolic syndrome, and particularly obesity remains essential for early detection and prevention of HS [8, 9]. Obesity accelerates HS by increasing fatty acid influx, activating lipogenesis, and reducing fatty acid oxidation, leading to fat accumulation in hepatocytes [10]. Diabetes may promote hepatic lipid synthesis through insulin resistance and reduced fatty acid oxidation, thereby facilitating HS progression [11]. Hyperlipidemia increases hepatic exposure to free fatty acids, promotes lipid synthesis and fat accumulation, and ultimately contributes to HS [12]. Genetic predisposition, lifestyle, and environmental factors also play roles [13]. Emerging evidence also suggests that inflammatory status is associated with HS, further supporting the need for early identification using multidimensional clinical indicators [14].

HS is a multifactorial and complex disease, and reliance on a single risk factor alone may not adequately capture its etiology, largely because the interrelationships between variables are difficult to evaluate using traditional methods [15]. Liver function tests (LFTs) are mainly used for assessing liver health by measuring blood parameters such as alanine aminotransferase (ALT) and aspartate aminotransferase (AST); however, reliance on LFT alone might lack specificity [16]. Ultrasound is widely used for initial HS evaluation owing to accessibility and low cost, yet it does have shortcomings in regard to sensitivity [17]. Single biomarkers may not achieve the level of comprehensiveness necessary to separate the presence of HS from different stages [18]. Machine learning facilitates HS prediction by integrating complex data from multiple sources, including clinical, laboratory, and imaging modalities [19]. Additionally, machine learning has a strong ability to identify hidden patterns in large datasets, thereby improving early detection and prediction.

Given the clinical burden of HS and the limitations of conventional single‐factor assessment, practical risk assessment tools based on routinely available clinical data are needed. Therefore, this study used multidimensional National Health and Nutrition Examination Survey (NHANES) data to develop and compare several machine learning models for predicting VCTE‐defined HS using non‐VCTE clinical variables. By integrating demographic characteristics, laboratory indices, and questionnaire‐based information, this study aimed to support early risk stratification and improve identification of individuals who may benefit from further elastography assessment.

## 2. Methods

### 2.1. Study Design and Participants

**FIGURE 1:**
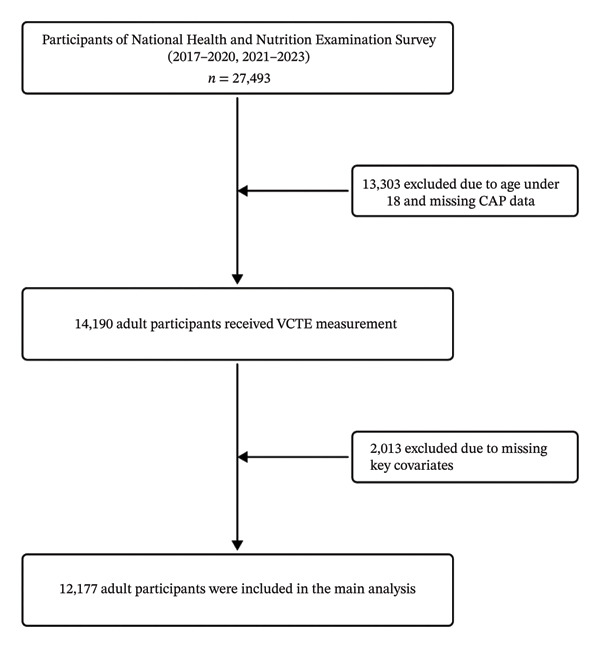
Flow diagram depicting the participant selection process.

This study used the NHANES database, which assesses the population’s nutritional and health status in the United States. NHANES is conducted in survey cycles through household interviews and examinations in mobile examination centers. Approval was obtained from the NCHS Research Ethics Review Board for the study, and all participants provided written informed consent. The study adhered to the Declaration of Helsinki. This study included 12,177 adult participants who completed elastography examinations from a total of 27,493 participants in two NHANES cycles, from 2017 to 2020 and from 2021 to 2023. Exclusion criteria for participants in the present study were as follows: (1) participants younger than 18 years; (2) lack of VCTE testing data; and (3) participants with more than 20% missing variables. Figure 1 depicts the screening procedure. Further details on NHANES are available at: https://wwwn.cdc.gov/nchs/nhanes.

### 2.2. Data Acquisition

Data were recorded in four different categories: demographics, examinations, laboratories, and questionnaires. Demographic data included gender, age, race, education, and the family poverty index ratio (PIR). Examination data included body mass index (BMI), liver stiffness measurement (LSM), and CAP. Laboratory data included high‐density lipoprotein (HDL) cholesterol, total cholesterol, platelets, red blood cells (RBC), hemoglobin, hematocrit, lymphocytes, monocytes, segmented neutrophils, glycohemoglobin, antihepatitis A virus (HAV), C‐reactive protein (CRP), cadmium, lead, manganese, mercury, and selenium blood levels. Questionnaire data included alcohol use, hypertension, hypercholesterolemia, diabetes, and smoking habits.

### 2.3. Definition of HS

Assessment of liver fat content using CAP values obtained from VCTE is a prevalent noninvasive technique. In the 2017–2020 and 2021–2023 NHANES cycles, VCTE examinations were performed using the FibroScan 502 V2 Touch with either the medium (M) or extra‐large (XL) probe. In this study, a CAP value ≥ 248 dB/m was used as the threshold for HS, which has been widely applied in practice for liver fat accumulation detection [20–22]. VCTE was used only to define HS, whereas the prediction models were developed using non‐VCTE clinical variables.

### 2.4. Statistical Analysis

Statistical analyses were performed with R 4.3.2 and Python 3.12. Variables were classified according to their type. Missing data were handled using multiple imputation by chained equations (MICE). Continuous variables were imputed using predictive mean matching, whereas categorical variables were imputed using regression‐based methods according to variable type. A total of 10 imputations were performed. Variables with missing values and their corresponding proportions of missingness are summarized in Table S1. A completed dataset was then used for subsequent feature selection and machine learning analyses. Continuous variables are expressed as mean ± standard deviation (SD) and compared using Welch’s *t*‐test or Mann–Whitney *U* test. Categorical variables are presented as counts and percentages and were compared using the chi‐square or Fisher’s exact test. *p* < 0.05 was considered statistically significant.

The Boruta algorithm and least absolute shrinkage and selection operator (LASSO) regression were applied for feature selection on the training set. Boruta, a random forest (RF)‐based wrapper method, identifies relevant variables by comparing the importance of original variables with that of randomized shadow variables over repeated iterations and classifies them as Confirmed, Rejected, or Tentative. In this study, the maximum number of iterations was set at 100, and variables confirmed by Boruta were retained for further analysis. LASSO regression was then applied for additional feature selection and dimensionality reduction. By introducing an L1 penalty, LASSO shrinks less informative coefficients toward zero, thereby reducing redundancy and minimizing overfitting. The optimal penalty parameter was determined by 10‐fold cross‐validation on the training set, and the lambda.1se value was selected to obtain a more parsimonious model. The combination of Boruta and LASSO leverages Boruta’s ability to identify relevant features and LASSO’s capacity to refine the feature set by reducing multicollinearity and enhancing interpretability, ensuring the stability and robustness of the feature set [23]. Spearman correlation analysis was performed among continuous variables to assess potential redundancy, with a threshold of |*r*| < 0.7 applied to ensure no highly redundant features were included. Ultimately, the selected predictors of HS were identified through the calculation of coefficients, odds ratios (OR), and 95% confidence intervals (CI) for each variable.

The dataset was then divided into a training set, with 70% of the data, and a validation set with 30% of the data. Prediction models were trained on six machine learning algorithms, specifically logistic regression (LR), RF, multilayer perceptron (MLP), support vector machine (SVM), K‐nearest neighbor (KNN), and extreme gradient boosting (XGBoost). Class imbalance was handled using model‐specific strategies, including class weighting, scale_pos_weight for XGBoost, and SMOTE for KNN. Hyperparameter optimization was performed only on the training set using grid search with 5‐fold cross‐validation. Regularization and early stopping were applied where appropriate to reduce overfitting. For each model, the optimal probability threshold was determined in the training set by maximizing Youden’s J statistic and was then fixed and applied to the validation set. Final model performance was evaluated in the validation set using AUC, accuracy, sensitivity, specificity, positive predictive value, and negative predictive value. Pairwise comparisons of AUC among the six machine learning models were performed using the DeLong test. SHapley Additive exPlanations (SHAP) was utilized in the interpretation of model predictions and to identify the most influential contributors to HS prediction. SHAP interaction analysis explored interactions between key variables. Subgroup analyses were conducted by age (< 65 vs. ≥ 65) and BMI (< 25 vs. ≥ 25) to assess XGBoost performance across subgroups.

## 3. Results

### 3.1. Characteristics of Participants

The total number of participants was 12,177, consisting of 5236 in the non‐HS and 6941 in the HS group (Table 1). Participants with HS were significantly older (52.93 ± 16.58 years vs. 46.76 ± 19.20 years), and a higher proportion were male (51.10% vs. 44.52%). Race was significantly different with higher representation of Mexican Americans and other Hispanics in the HS group (12.52% vs. 7.58% and 10.66% vs. 9.55%). The HS group had a higher proportion of participants with less than 9th grade education, whereas the proportion with PIR < 1.3 was slightly lower than that in the non‐HS group. The two groups differed significantly in BMI, HDL cholesterol, total cholesterol, platelet count, RBC, hemoglobin, hematocrit, lymphocytes, monocytes, segmented neutrophils, glycohemoglobin, anti‐HAV, CRP, cadmium, manganese, mercury, selenium, smoking, hypertension, hypercholesterolemia, and diabetes, but not in alcohol consumption (*p* = 0.13) or blood lead level (*p* = 0.83).

**TABLE 1:** Baseline characteristics of included participants.

Variable	Nonhepatic steatosis (*n* = 5236)	Hepatic steatosis (*n* = 6941)	*p* value
Age	46.76 ± 19.20	52.93 ± 16.58	< 0.01
Gender			< 0.01
Male	2331 (44.52%)	3547 (51.10%)	
Female	2905 (55.48%)	3394 (48.90%)	
Race			< 0.01
Mexican American	397 (7.58%)	869 (12.52%)	
Other Hispanic	500 (9.55%)	740 (10.66%)	
Non‐Hispanic White	2305 (44.02%)	3099 (44.65%)	
Non‐Hispanic Black	1235 (23.59%)	1249 (17.99%)	
Other	799 (15.26%)	984 (14.18%)	
Education			< 0.01
Less than 9th grade	222 (4.24%)	459 (6.61%)	
9–11th grade	435 (8.31%)	652 (9.39%)	
High school graduate	1167 (22.29%)	1658 (23.89%)	
Some College or AA degree	1657 (31.65%)	2353 (33.90%)	
College graduate or above	1752 (33.51%)	1813 (26.11%)	
PIR			< 0.01
Less than 1.3	1194 (22.80%)	1467 (21.14%)	
1.3–3.5	1642 (31.36%)	2403 (34.62%)	
Greater than 3.5	1746 (33.35%)	2166 (31.21%)	
BMI	25.84 ± 5.19	32.64 ± 7.25	< 0.01
HDL Cholesterol	58.42 ± 15.25	50.24 ± 14.16	< 0.01
Total Cholesterol	182.30 ± 39.14	189.12 ± 41.33	< 0.01
Platelet	245.90 ± 63.27	253.37 ± 66.82	< 0.01
RBC	4.64 ± 0.48	4.78 ± 0.49	< 0.01
Hemoglobin	13.83 ± 1.48	14.14 ± 1.51	< 0.01
Hematocrit	41.11 ± 4.04	41.98 ± 4.07	< 0.01
Lymphocyte	2.03 ± 1.18	2.22 ± 0.81	< 0.01
Monocyte	0.53 ± 0.18	0.58 ± 0.20	< 0.01
Segmented neutrophils	3.87 ± 1.57	4.33 ± 1.63	< 0.01
Glycohemoglobin	5.50 ± 0.75	6.00 ± 1.21	< 0.01
Antihepatitis A virus	1.53 ± 0.51	1.55 ± 0.50	0.03
CRP	2.81 ± 6.80	4.69 ± 8.37	< 0.01
Cadmium	0.46 ± 0.53	0.43 ± 0.54	< 0.01
Lead	1.09 ± 1.28	1.10 ± 1.20	0.83
Manganese	9.41 ± 3.51	9.83 ± 3.68	< 0.01
Mercury	1.35 ± 2.30	1.25 ± 2.26	0.02
Selenium	183.01 ± 29.48	185.88 ± 27.38	< 0.01
Alcohol consumption	4899 (93.56%)	6532 (94.11%)	0.13
Smoking	1943 (37.11%)	2990 (43.08%)	< 0.01
Hypertension	1337 (25.53%)	3053 (43.99%)	< 0.01
Hypercholesterolemia	1504 (28.72%)	2959 (42.63%)	< 0.01
Diabetes	336 (6.42%)	1302 (18.76%)	< 0.01

*Note:* PIR, family poverty index ratio.

Abbreviations: BMI, body mass index; CRP, C‐reactive protein; HDL, high‐density lipoprotein; RBC, red blood cell.

### 3.2. Feature Selection

**FIGURE 2:**
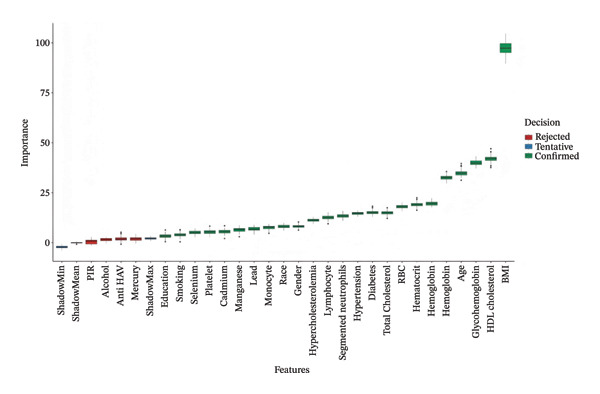
Feature selection results from the Boruta algorithm.

**FIGURE 3:**
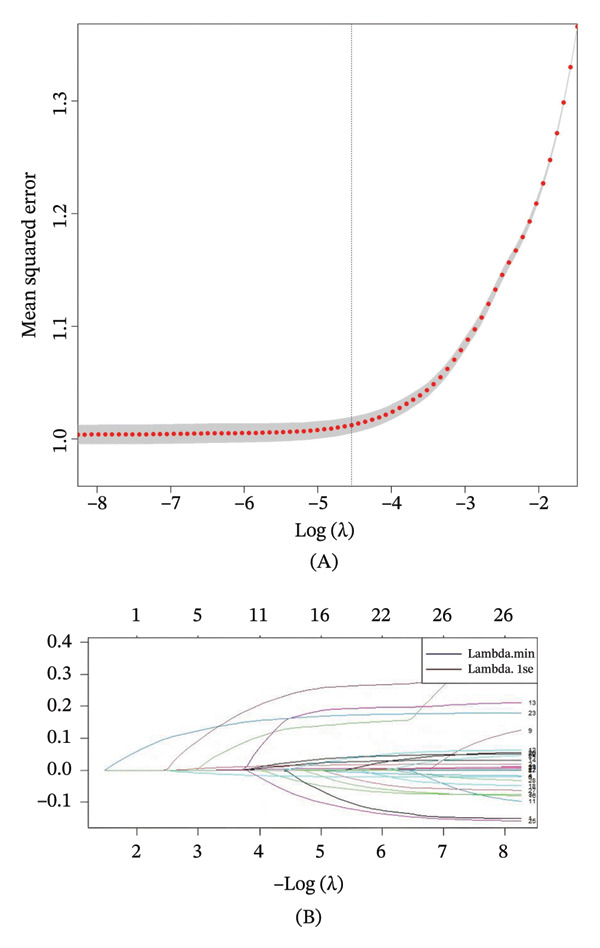
Results from the LASSO regression analysis. (A) Feature screening based on the LASSO regression analysis and (B) LASSO regression analysis screening variable trajectories.

**FIGURE 4:**
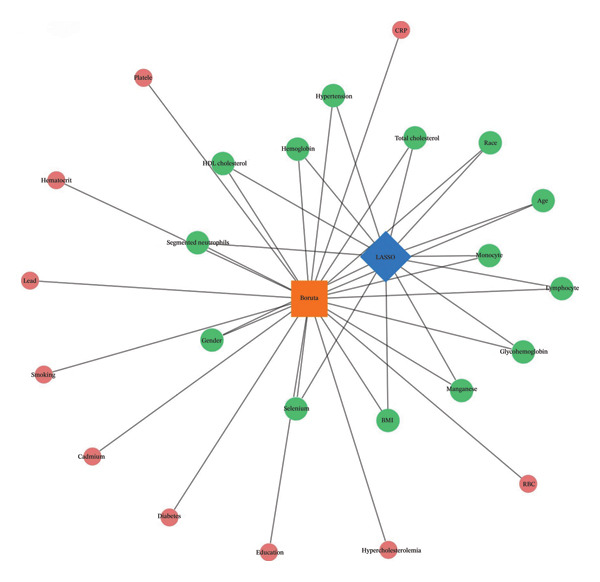
Common predictors between Boruta algorithm and LASSO regression analysis.

The Boruta algorithm identified 24 significant features for HS independently, such as BMI, HDL‐cholesterol, glycohemoglobin, age, CRP, hemoglobin, hematocrit, RBC, total‐cholesterol, and lymphocyte, etc. (Figure 2). In contrast, anti‐HAV, mercury, alcohol consumption, and PIR were eliminated as noninformative variables (Table S2). LASSO regression identified 14 significant features, including gender, age, race, total cholesterol, hemoglobin, lymphocyte, monocyte, and segmented neutrophils, in which the selection was done on coefficients at lambda.1se (Figure 3). Among them, glycohemoglobin, monocyte, BMI, and hemoglobin were found to be the most significant features (Figure S1 and Table S3). The intersection of features selected by Boruta and LASSO yielded 14 stable predictors: age, BMI, gender, glycohemoglobin, HDL cholesterol, hemoglobin, hypertension, lymphocyte, manganese, monocyte, race, segmented neutrophils, selenium, and total cholesterol (Figure 4). No strong pairwise correlations were found among continuous variables (all |r| < 0.3), suggesting minimal redundancy.

### 3.3. Comparison of Predictive Performance Among Six Machine Learning Models

**FIGURE 5:**
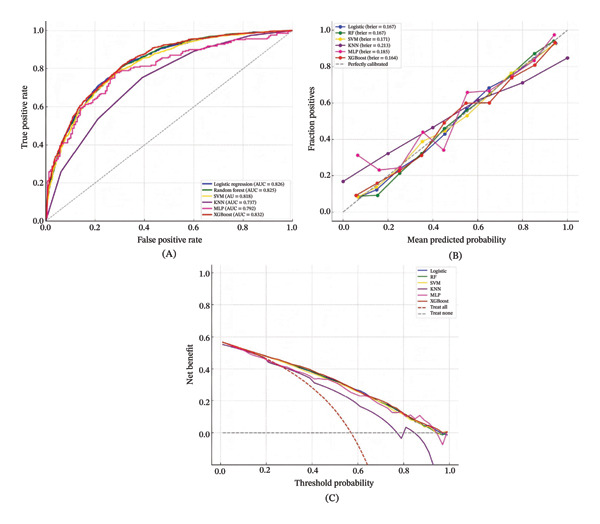
Predicted performance of different machine learning models. (A) Comparison of ROC curve based on validation set; (B) comparison of calibration curves; and (C) comparison of decision curves.

Based on these selected features, we trained predictive models using six different machine learning algorithms. Table 2 reports the predictive performance of different machine learning models. XGBoost achieved the highest AUC of 0.832 (0.819–0.845), with an accuracy of 77.1%, sensitivity of 80.3%, specificity of 72.8%, PPV of 80.1%, NPV of 73.0%, and an optimal threshold of 0.512. The other models also showed acceptable predictive performance, with the LR model having an AUC of 0.826 (0.811–0.838), the RF model having an AUC of 0.825 (0.813–0.839), the SVM model having an AUC of 0.818 (0.805–0.832), the MLP having an AUC of 0.792 (0.746–0.833), and the KNN having an AUC of 0.737 (0.721–0.752). Pairwise DeLong tests showed that XGBoost significantly outperformed KNN, MLP, and SVM in terms of AUC, while LR and RF showed comparable performance (Table S4). Decision curve analysis showed XGBoost to produce the highest net benefit among all models, while the calibration curve also confirmed the superior calibration of XGBoost through the lowest Brier score (0.164), revealing a high concordance of predicted probabilities and true events (Figure 5). The confusion matrix also confirmed the same with 1693 true positives, 1125 true negatives, 420 false positives, and 416 false negatives (Figure S2).

**TABLE 2:** Comparison of prediction performance of six machine learning models.

Model	AUC (95% CI)	Accuracy	Sensitivity	Specificity	PPV	NPV	Threshold
Logistic Regression	0.826 (0.811–0.838)	0.761	0.814	0.690	0.777	0.736	0.517
Random Forest	0.825 (0.813–0.839)	0.759	0.809	0.693	0.760	0.747	0.535
Extreme Gradient Boosting	0.832 (0.819–0.845)	0.771	0.803	0.728	0.801	0.730	0.512
K‐Nearest Neighbors	0.737 (0.721–0.752)	0.691	0.753	0.609	0.719	0.651	0.600
Multilayer Perceptron	0.792 (0.746–0.833)	0.754	0.780	0.720	0.787	0.711	0.487
Support Vector Machine	0.818 (0.805–0.832)	0.751	0.782	0.710	0.782	0.710	0.547

*Note:* AUC, area under the receiver operating characteristic curve.

Abbreviations: CI, confidence interval; NPV, negative predictive value; PPV, positive predictive value.

### 3.4. SHAP‐Based Model Interpretability Analysis

The SHAP summary plot and SHAP bar plot both showed BMI to be the strongest predictor of HS, having the highest impact on the output of the model. Glycohemoglobin, age, and HDL cholesterol were ranked second, third, and fourth, respectively (Figure 6A and Figure 6B). Higher values of BMI, glycohemoglobin, and age were associated with increased predicted risk for HS. In contrast, lower HDL cholesterol was associated with higher risk (Figure 6C). Analysis of categorical variables showed that gender had minimal impact on the model, whereas race contributed slightly more variability (Figure 6D). XGBoost feature importance analysis based on Gain also supports these findings, with glycohemoglobin (Gain = 0.506), BMI (0.079), and hemoglobin (0.068) contributing substantially to the model (Table S5). Table S6 presents the results of the SHAP interaction analysis, where the strongest interaction effects were observed between age and BMI.

FIGURE 6SHAP diagram for predicting hepatic steatosis using the XGBoost model. (A) SHAP summary plot of feature importance; (B) SHAP bar plot of feature importance; (C) SHAP trend chart of continuous variable changes; and (D) SHAP box plots of categorical variables.

**Figure:**
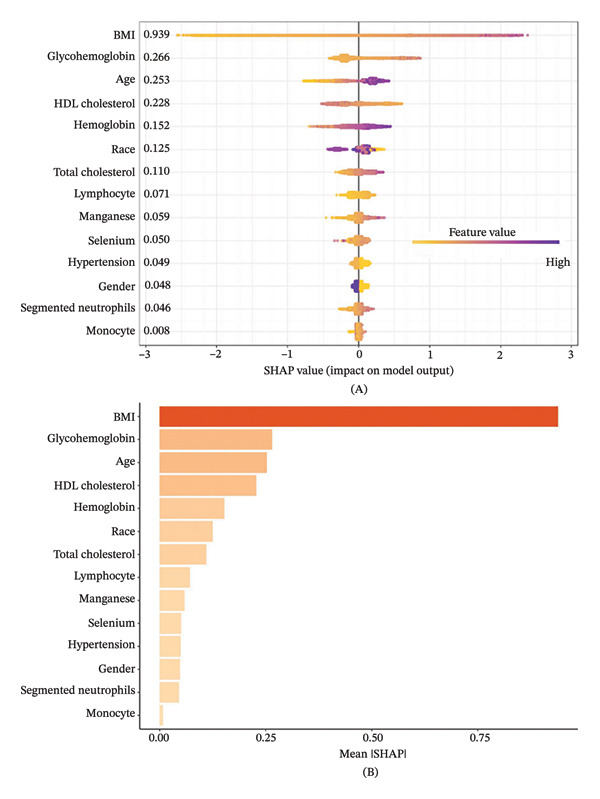

**Figure:**
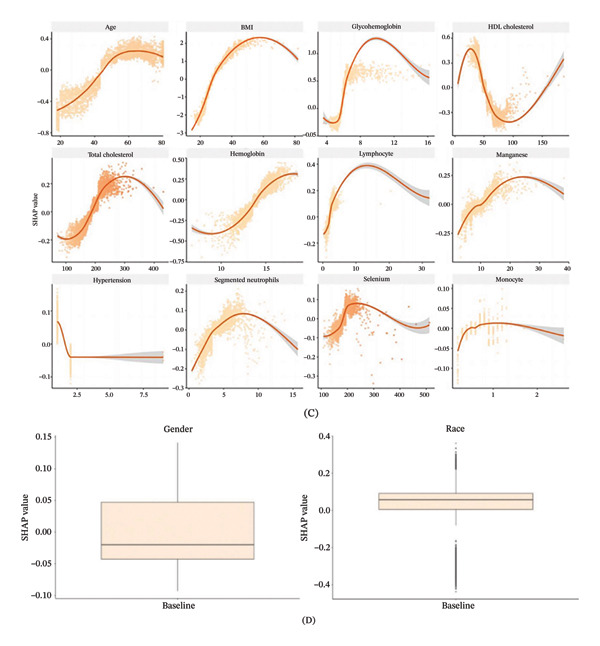

### 3.5. Subgroup Analysis

Subgroup analyses were conducted by age (< 65 vs. ≥ 65) and BMI (< 25 vs. ≥ 25). For age, the AUC was 0.849 (95% CI: 0.835–0.864) for individuals < 65 and 0.772 (95% CI: 0.741–0.804) for individuals ≥ 65, with corresponding accuracies of 0.769 and 0.711, sensitivities of 0.755 and 0.765, and specificities of 0.787 and 0.623. For BMI, the AUC was 0.734 (95% CI: 0.694–0.773) for BMI < 25 and 0.773 (95% CI: 0.755–0.792) for BMI ≥ 25, with accuracies of 0.797 and 0.739, sensitivities of 0.098 and 0.830, and specificities of 0.991 and 0.533 (Table S7).

## 4. Discussion

This study investigated predictors of HS using data from two NHANES cycles. A total of 14 key features were identified, including age, BMI, gender, glycohemoglobin, HDL cholesterol, hemoglobin, hypertension, lymphocyte, manganese, monocyte, race, segmented neutrophils, selenium, and total cholesterol. Based on these variables, six machine learning models were developed and compared, among which XGBoost showed the best overall predictive performance. SHAP analysis highlighted BMI, glycohemoglobin, age, and HDL cholesterol as the most influential predictors. The subgroup analysis indicated that the model’s predictive performance varied across age and BMI groups, performing slightly better in younger individuals and showing higher sensitivity in participants with higher BMI. These findings suggest that routinely available non‐VCTE variables may provide useful information for the early identification and risk stratification of HS in a large population‐based sample.

Numerous studies have investigated the use of machine learning models for the prediction of HS. One study built an LR model based on potential liver donors, incorporating multidimensional data such as clinical, laboratory, and imaging variables, and achieved high predictive accuracy for HS [24]. Another study indicated that each of three machine learning classifiers based on body composition indices and DXA‐derived variables had high predictive power, with AUC values from 0.83 to 0.87 [25]. Researchers also developed an XGBoost‐based model incorporating clinical, laboratory, and traditional Chinese medicine data to predict HS in patients at high metabolic risk, achieving an AUC of 0.82 [26]. Yan et al. established the Environmental Pollution Exposure Index (EPEI) using 2015–2016 NHANES data and developed a stacked ensemble model with good predictive power for HS [27]. In addition, researchers used LASSO plus RF in 4227 NHANES 2021‐2023 participants and achieved an overall validation AUC of 0.80 [7]. Our findings further support the use of machine learning for predicting HS. In contrast, our study combines routine clinical variables with interpretable machine learning methods to predict VCTE‐defined HS. Given the limited access to elastography in some settings, we propose a framework using demographic, laboratory, and questionnaire data for early risk assessment. By comparing multiple algorithms and applying SHAP interpretation, we enhance understanding of model performance and the contribution of key variables. This approach improves the model’s clinical interpretability, making it more useful in clinical settings, particularly for clinicians who require transparent, understandable results to support decision‐making. This increased interpretability could lead to more effective screening, early risk stratification, and referral decisions, which are crucial in real‐world clinical applications where elastography may not always be available.

We identified 14 key predictors of HS, including BMI, glycohemoglobin, HDL cholesterol, and hemoglobin. Glycohemoglobin indicates long‐term blood glucose control, and elevated levels are usually associated with insulin resistance, thereby increasing the risk of HS through promotion of hepatic fat accumulation [28]. HDL cholesterol may be protective against liver fat accumulation, whereas low HDL cholesterol is associated with a higher risk of fatty liver, possibly because impaired reverse cholesterol transport may promote HS [29]. Hemoglobin may be linked to HS in the context of metabolic dysfunction, and this association may partly relate to disturbances in iron metabolism and oxidative stress [30]. SHAP analysis of the XGBoost model further highlighted the importance of these predictors in HS prediction, particularly metabolic and hematological features. BMI showed the greatest overall influence in the SHAP analysis, consistent with its well‐established role in predicting metabolic disorders and fatty liver disease. The difference between SHAP and Gain rankings suggests that these metrics can produce different feature‐ranking patterns, because SHAP measures each feature’s contribution to the model output, while Gain quantifies the reduction in loss during tree splitting [31]. Thus, SHAP and Gain provide complementary but distinct perspectives on feature importance. Age, also identified as an important predictor, has a well‐documented association with HS in clinical studies. The interaction analysis between age and BMI revealed that the impact of BMI on HS is stronger in older individuals, suggesting that aging exacerbates the effect of BMI on health outcomes due to reduced metabolic function, increased fat accumulation, and decreased insulin sensitivity with age [32, 33].

This study has several limitations. First, the analysis used only NHANES data, and no external validation was performed, which may limit generalizability. Second, because of the cross‐sectional design, causal relationships between predictors and HS cannot be established. Third, HS was defined using the CAP threshold rather than liver biopsy, and CAP cannot distinguish the underlying etiology of steatosis. Therefore, alcohol‐related steatotic liver disease could not be fully excluded. Fourth, differences in sample design between NHANES cycles and exclusion of participants with missing VCTE data may introduce selection bias. Additionally, model performance may have been influenced by data quality, feature selection procedures, and parameter tuning, and unmeasured confounding factors, including medication use such as statins or thiazolidinediones, may have affected both steatosis status and laboratory variables. Future research could focus on exploring novel methodological approaches to improve model interpretability and predictive performance, as well as conducting external validation in independent, diverse cohorts.

## 5. Conclusion

This study developed a machine learning‐based risk prediction model for HS, in which XGBoost achieved the highest AUC. BMI, age, glycohemoglobin, and HDL cholesterol were identified as clinically useful features for early detection and risk stratification of HS. Although the model showed reasonable performance, further validation and optimization are needed before it can be widely applied across different populations.

## Funding

This study was supported by the Research on the Construction and Application of an Artificial Intelligence‐Based Low‐Cost Medical Internet of Things Shared Platform System (No. 20250326).

## Ethics Statement

This survey study was approved by the National Center for Health Statistics Ethics Review Board of the US. The consent form was signed by participants in the survey.

## Conflicts of Interest

The authors declare no conflicts of interest.

## Supporting Information

Additional supporting information can be found online in the Supporting Information section.

## Supporting information


**Supporting Information** . Additional information is provided in Supporting Tables S1–S7 and Supporting Figures S1‐S2, including missing data summaries, feature selection results, model comparison, SHAP interaction analysis, subgroup analysis, and validation results. Table S1. Summary of variables with missing values. Table S2. Results of Boruta algorithm for feature selection. Table S3. Results of LASSO regression analysis for selected features. Table S4. Pairwise DeLong test results for AUC comparisons among the six machine learning models. Table S5. XGBoost Feature Importance based on Gain. Table S6. Top 20 feature interactions by mean absolute SHAP interaction strength. Table S7. Subgroup Analysis Results Based on Age and BMI. Figure S1. LASSO regression coefficients of selected variables. Figure S2. The confusion matrix for internal validation of XGBoost model.

## Data Availability

The original contributions presented in the study are included in the article and Supporting Information. Further inquiries can be directed to the corresponding author.
