# Supplementary material for: Machine Learning and SHAP Value Interpretation for Predicting Hepatic Steatosis Using Vibration‐Controlled Transient Elastography
Source: Int J Endocrinol. 2026 Jun 18;2026:3395722. doi: 10.1155/ije/3395722 (PMC13277773; doi:10.1155/ije/3395722)

**Supplemental files**

**File directory**

**Table S1. Summary of variables with missing values**

**Table S2. Results of Boruta algorithm for feature selection**

**Table S3. Results of LASSO regression analysis for selected features**

**Table S4. Pairwise DeLong test results for AUC comparisons among the six machine learning models.**

**Table S5. XGBoost Feature Importance based on Gain**

**Table S6. Top 20 feature interactions by mean absolute SHAP interaction strength**

**Table S7. Subgroup Analysis Results Based on Age and BMI**

**Figure S1. LASSO regression coefficients for selected features**

**Figure S2. The confusion matrix for internal validation of XGBoost mode**l

**Table S1. Summary of variables with missing values**

| **Variable** | **Missing Values** | **Missing Ratio (%)** | **Imputation Method** |
| --- | --- | --- | --- |
| Education | 487 | 4.00 | Regression-based imputation |
| PIR | 1559 | 12.80 | Predictive mean matching |
| HDL Cholesterol | 362 | 2.97 | Predictive mean matching |
| Total Cholesterol | 362 | 2.97 | Predictive mean matching |
| Platelet | 20 | 0.16 | Predictive mean matching |
| RBC | 20 | 0.16 | Predictive mean matching |
| Hemoglobin | 20 | 0.16 | Predictive mean matching |
| Hematocrit | 20 | 0.16 | Predictive mean matching |
| Lymphocyte | 30 | 0.25 | Predictive mean matching |
| Monocyte | 30 | 0.25 | Predictive mean matching |
| Segmented neutrophils | 30 | 0.25 | Predictive mean matching |
| Glycohemoglobin | 24 | 0.20 | Predictive mean matching |
| Anti HAV | 294 | 2.41 | Regression-based imputation |
| CRP | 351 | 2.88 | Predictive mean matching |
| BMI | 84 | 0.69 | Predictive mean matching |
| Alcohol | 408 | 3.35 | Regression-based imputation |
| Smoking | 1 | 0.01 | Regression-based imputation |
| Abbreviations: PIR, family poverty index ratio; HDL, high-density lipoprotein; RBC, Red blood cell; CRP, C-reactive protein; BMI, body mass index. | | | |

**Table S2. Results of Boruta algorithm for feature selection**

| **Feature** | **MeanImp** | **MedianImp** | **MinImp** | **MaxImp** | **NormHits** | **Decision** |
| --- | --- | --- | --- | --- | --- | --- |
| Body mass index | 97.27 | 97.37 | 89.60 | 104.62 | 1.0 | Confirmed |
| HDL Cholesterol | 41.97 | 42.09 | 37.67 | 47.02 | 1.0 | Confirmed |
| Glycohemoglobin | 40.01 | 39.87 | 37.21 | 43.33 | 1.0 | Confirmed |
| Age | 34.82 | 34.78 | 31.25 | 39.64 | 1.0 | Confirmed |
| CRP | 32.53 | 32.58 | 29.73 | 35.61 | 1.0 | Confirmed |
| Hemoglobin | 19.58 | 19.59 | 17.66 | 22.28 | 1.0 | Confirmed |
| Hematocrit | 19.07 | 19.07 | 16.25 | 22.46 | 1.0 | Confirmed |
| RBC | 18.07 | 17.97 | 15.81 | 20.19 | 1.0 | Confirmed |
| Diabetes | 15.13 | 15.03 | 13.00 | 18.22 | 1.0 | Confirmed |
| Total Cholesterol | 14.97 | 15.04 | 12.19 | 17.49 | 1.0 | Confirmed |
| Hypertension | 14.67 | 14.78 | 12.98 | 16.18 | 1.0 | Confirmed |
| Segmented neutrophils | 13.48 | 13.39 | 10.97 | 15.95 | 1.0 | Confirmed |
| Lymphocyte | 12.59 | 12.60 | 9.51 | 15.31 | 1.0 | Confirmed |
| Hypercholesterolemia | 11.29 | 11.30 | 9.33 | 12.98 | 1.0 | Confirmed |
| Gender | 8.18 | 8.20 | 6.31 | 10.36 | 1.0 | Confirmed |
| Race | 8.11 | 8.04 | 6.04 | 9.97 | 1.0 | Confirmed |
| Monocyte | 7.65 | 7.83 | 4.70 | 9.36 | 1.0 | Confirmed |
| Lead | 6.92 | 6.74 | 4.07 | 8.94 | 1.0 | Confirmed |
| Manganese | 6.49 | 6.64 | 3.02 | 8.81 | 1.0 | Confirmed |
| Cadmium | 5.56 | 5.55 | 2.12 | 8.50 | 1.0 | Confirmed |
| Platelet | 5.32 | 5.40 | 2.69 | 8.28 | 1.0 | Confirmed |
| Selenium | 5.16 | 5.05 | 2.76 | 7.20 | 1.0 | Confirmed |
| Smoking | 3.99 | 3.97 | 0.48 | 6.49 | 1.0 | Confirmed |
| Education | 3.31 | 3.33 | 0.55 | 6.39 | 0.8 | Confirmed |
| Anti HAV | 1.87 | 1.81 | -0.76 | 5.23 | 0.4 | Rejected |
| Mercury | 1.86 | 1.99 | -0.45 | 4.36 | 0.4 | Rejected |
| Alcohol | 1.47 | 1.59 | -0.38 | 2.87 | 0.1 | Rejected |
| PIR | 0.50 | 0.78 | -1.32 | 2.89 | 0.0 | Rejected |
| Abbreviations: BMI, body mass index; HDL, high-density lipoprotein; CRP, C-reactive protein; RBC, Red blood cell; PIR, family poverty index ratio. | | | | | | |

**Table S3. Results of LASSO regression analysis for selected features**

| **Feature** | **Coefficient** | **OR** | **95% CI** |
| --- | --- | --- | --- |
| Gender | -0.165 | 0.848 | 0.757-0.949 |
| Age | 0.018 | 1.019 | 1.016-1.022 |
| Race | -0.087 | 0.916 | 0.881-0.953 |
| HDL Cholesterol | -0.021 | 0.979 | 0.976-0.983 |
| Total Cholesterol | 0.006 | 1.006 | 1.005-1.007 |
| Hemoglobin | 0.153 | 1.165 | 1.123-1.208 |
| Lymphocyte | 0.077 | 1.080 | 1.011-1.153 |
| Monocyte | 0.208 | 1.231 | 0.931-1.628 |
| Segmented neutrophils | 0.036 | 1.037 | 1.004-1.071 |
| Glycohemoglobin | 0.295 | 1.343 | 1.267-1.423 |
| Manganese | 0.053 | 1.054 | 1.041-1.068 |
| Selenium | 0.002 | 1.0023 | 1.000-1.004 |
| BMI | 0.178 | 1.195 | 1.184-1.206 |
| Hypertension | -0.154 | 0.858 | 0.785-0.937 |
| Abbreviations: OR, odds ratio; CI, confidence interval; HDL, high-density lipoprotein; BMI, body mass index. | | | |

**Table S4. Pairwise DeLong test results for AUC comparisons among the six machine learning models.**

| **Model 1** | **Model 2** | **AUC 1** | **AUC 2** | **AUC_Difference** | **Z_stat** | **P_value** |
| --- | --- | --- | --- | --- | --- | --- |
| Support Vector Machine | K-Nearest Neighbors | 0.818 | 0.737 | 0.081 | 19.715 | P < 0.001 |
| K-Nearest Neighbors | Extreme Gradient Boosting | 0.737 | 0.832 | -0.095 | -21.978 | P < 0.001 |
| K-Nearest Neighbors | Multi-layer Perceptron | 0.737 | 0.792 | -0.055 | -13.415 | P < 0.001 |
| Random Forest | K-Nearest Neighbors | 0.825 | 0.737 | 0.088 | 21.136 | P < 0.001 |
| Logistic Regression | K-Nearest Neighbors | 0.826 | 0.737 | 0.089 | 20.479 | P < 0.001 |
| Support Vector Machine | Multi-layer Perceptron | 0.818 | 0.792 | 0.026 | 7.953 | P < 0.001 |
| Logistic Regression | Support Vector Machine | 0.826 | 0.818 | 0.008 | 3.074 | 0.002 |
| Random Forest | Extreme Gradient Boosting | 0.825 | 0.832 | -0.007 | -2.552 | 0.027 |
| Random Forest | Support Vector Machine | 0.825 | 0.818 | 0.007 | 2.214 | 0.021 |
| Multi-layer Perceptron | Extreme Gradient Boosting | 0.792 | 0.832 | -0.040 | -9.234 | P < 0.001 |
| Logistic Regression | Extreme Gradient Boosting | 0.826 | 0.832 | -0.006 | -2.021 | 0.043 |
| Logistic Regression | Multi-layer Perceptron | 0.826 | 0.792 | 0.034 | 8.665 | P < 0.001 |
| Support Vector Machine | Extreme Gradient Boosting | 0.818 | 0.832 | -0.014 | -5.890 | P < 0.001 |
| Random Forest | LogisticRegression | 0.825 | 0.826 | -0.001 | -0.271 | 0.787 |
| Random Forest | Multi-layer Perceptron | 0.825 | 0.792 | 0.033 | 7.977 | P < 0.001 |
| Abbreviations: AUC, area under the receiver operating characteristic curve. | | | | | | |

**Table S5. XGBoost Feature Importance based on Gain**

| **Feature** | **Gain** |
| --- | --- |
| Glycohemoglobin | 0.506 |
| Monocyte | 0.086 |
| BMI | 0.079 |
| Hemoglobin | 0.068 |
| Hypertension | 0.053 |
| Race | 0.043 |
| Manganese | 0.035 |
| HDL cholesterol | 0.034 |
| Segmented neutrophils | 0.031 |
| Age | 0.026 |
| Gender | 0.022 |
| Lymphocyte | 0.007 |
| Total cholesterol | 0.006 |
| Selenium | 0.005 |
| Abbreviations: BMI, body mass index; HDL, high-density lipoprotein. | |

**Table S6. Top 20 feature interactions by mean absolute SHAP interaction strength**

| **Feature 1** | **Feature 2** | **MeanAbsInteraction** |
| --- | --- | --- |
| Age | BMI | 0.034 |
| Glycohemoglobin | BMI | 0.032 |
| BMI | Non-Hispanic Black | 0.021 |
| HDL Cholesterol | BMI | 0.019 |
| Hemoglobin | BMI | 0.016 |
| Age | Total Cholesterol | 0.014 |
| BMI | Other Race | 0.013 |
| Segmented neutrophils | BMI | 0.013 |
| Age | Glycohemoglobin | 0.011 |
| HDL Cholesterol | Glycohemoglobin | 0.010 |
| HDL Cholesterol | Total Cholesterol | 0.009 |
| Age | HDL Cholesterol | 0.009 |
| HDL Cholesterol | Hemoglobin | 0.008 |
| Total Cholesterol | BMI | 0.007 |
| BMI | Female | 0.006 |
| Total Cholesterol | Glycohemoglobin | 0.005 |
| Manganese | BMI | 0.005 |
| Hemoglobin | Glycohemoglobin | 0.005 |
| Hemoglobin | Lymphocyte | 0.005 |
| HDL Cholesterol | Non-Hispanic Black | 0.005 |
| Abbreviations: BMI, body mass index; HDL, high-density lipoprotein. | | |

**Table S7. Subgroup Analysis Results Based on Age and BMI**

| **Group** | **Samples** | **AUC (95% CI)** | **Accuracy** | **Sensitivity** | **Specificity** |
| --- | --- | --- | --- | --- | --- |
| Age |  |  |  |  |  |
| < 65 | 2718 | 0.849 (0.835 - 0.864) | 0.769 | 0.755 | 0.787 |
| ≥ 65 | 936 | 0.772 (0.741 - 0.804) | 0.711 | 0.765 | 0.623 |
| BMI |  |  |  |  |  |
| < 25 | 947 | 0.734 (0.694 - 0.773) | 0.797 | 0.098 | 0.991 |
| ≥ 25 | 2707 | 0.773 (0.755 - 0.792) | 0.739 | 0.83 | 0.533 |
| Abbreviations: AUC, area under the receiver operating characteristic curve; CI, confidence interval; BMI, body mass index. | | | | | |

**Figure S1. LASSO regression coefficients for selected features**


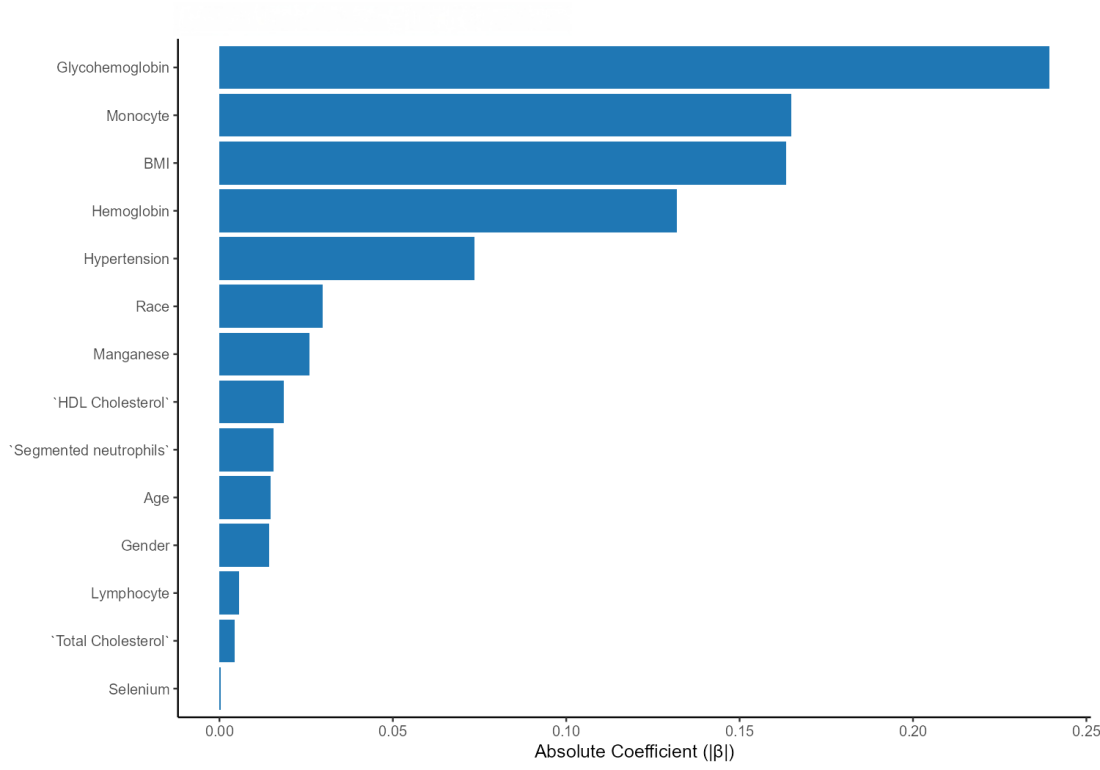


**Figure S2. The confusion matrix for internal validation of XGBoost model**


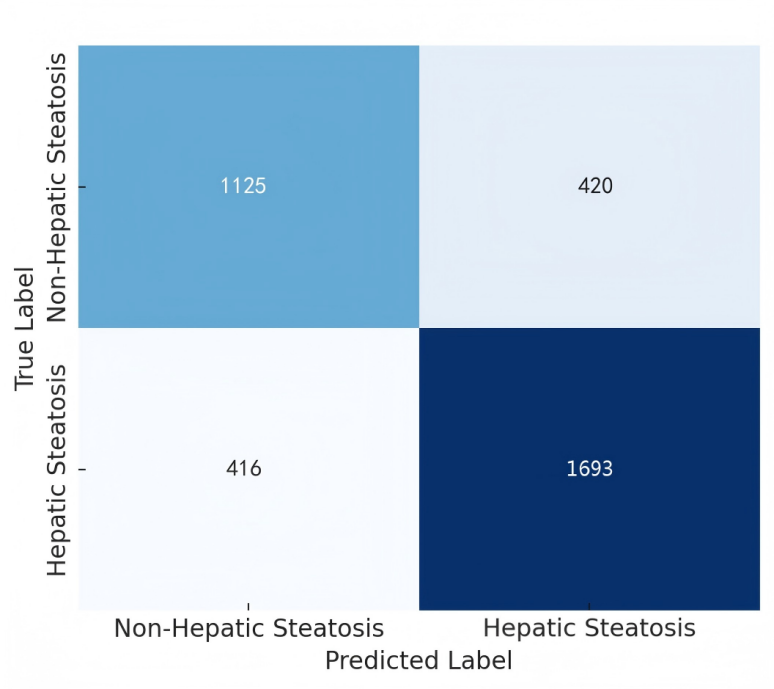

Supplement: Supplementary file 1 — Supporting Information . Additional information is provided in Supporting Tables S1–S7 and Supporting Figures S1‐S2, including missing data summaries, feature selection results, model comparison, SHAP interaction analysis, subgroup analysis, and validation results. Table S1. Summary of variables with missing values. Table S2. Results of Boruta algorithm for feature selection. Table S3. Results of LASSO regression analysis for selected features. Table S4. Pairwise DeLong test results for AUC comparisons among the six machine learning models. Table S5. XGBoost Feature Importance based on Gain. Table S6. Top 20 feature interactions by mean absolute SHAP interaction strength. Table S7. Subgroup Analysis Results Based on Age and BMI. Figure S1. LASSO regression coefficients of selected variables. Figure S2. The confusion matrix for internal validation of XGBoost model. [file IJE-2026-3395722-s001.docx]
